# Fluoridation in Chile: historical context, current programs, challenges, and future directions

**DOI:** 10.3389/froh.2026.1844295

**Published:** 2026-05-15

**Authors:** Rodrigo Mariño, Melisa Münzenmayer, Carlos Zaror

**Affiliations:** 1 Melbourne Dental School, The University of Melbourne, Melbourne, VIC, Australia; 2 Center for Research in Epidemiology, Economics and Oral Public Health (CIEESPO), Faculty of Dentistry, Universidad de La Frontera, Temuco, Chile; 3 Monash Health, Dental Services, Monash Health Dental Services, Melbourne, VIC, Australia; 4 Melbourne School of Population and Global Health, The University of Melbourne, Melbourne, VIC, Australia; 5 Instituto de Salud Pública, Facultad de Medicina, Universidad Austral de Chile, Valdivia, Chile; 6 Department of Pediatric Dentistry and Orthodontics, Faculty of Dentistry, Universidad de La Frontera, Temuco, Chile

**Keywords:** Chile, community water fluoridation, fluoride programs, oral health, public health policy

## Abstract

As part of the Fluoride Across the World effort, this manuscript aims to examine the history, current programs, challenges, and future directions of fluoridation initiatives in Chile. This analysis describes the evolution of fluoridation practices, including early adoption, the various fluoride programs and delivery methods implemented, community engagement efforts, and the challenges of implementation. Key milestones in governmental policies and health organization efforts will also be discussed. The primary objective of this review was to assess the effectiveness, safety, and cost-effectiveness of oral health fluoride policies and programs implemented in Chile. The study highlights the historical significance of the adoption of water fluoridation in Chile, beginning in the 1950s, and its expansion, driven by government support, leading to its widespread implementation. Current fluoridation initiatives, including water, and milk, are described. The paper underscores the importance of fluoridation as a public health strategy for preventing dental caries and promoting oral health. It identifies ongoing challenges and suggests future directions, including the integration of new technologies to support community education and monitoring, thereby contributing to the discourse on fluoride's role in public health policy. Fluorides, particularly in the context of water and health, can be linked to several of the United Nations Sustainable Development Goals (SDGs). Within this background, the review identifies ongoing challenges such as public misinformation and regulatory inconsistencies that obstruct the effective maintenance and implementation of fluoridation programs. Thus, this analysis would be relevant for policymakers and public health practitioners as it provides valuable insights and suggests future directions aimed at improving oral health outcomes through effective fluoridation strategies, ultimately enhancing community health and wellbeing.

## 1 Introduction

In the 19th century, German scientists first reported some protective effects from fluoride in animals (1), however, the early adoption of water fluoridation can be traced back to the 1940s, following significant contributions from researchers such as Dr. Frederick McKay and Dr. H. Trendley Dean (2). Their investigations into the prevalence of dental fluorosis and caries in Colorado Springs led to the identification of the protective effects of fluoride (2). In 1945, Grand Rapids, Michigan became the first city to implement a community water fluoridation program, marking a pivotal moment in public health policy (2). The success of this initiative soon led to its adoption by other cities, leading to a nationwide movement. By 2019, approximately 74% of the U.S. population served by community water systems received fluoridated water (3).

Fluoride's role in oral health has been extensively studied since the early 20th century. For over 80 years, laboratory, clinical, and community research have investigated the use of fluorides in the prevention of dental caries. Its application is widely accepted as a safe, effective, efficient, and appropriate mechanism for preventing dental decay (4, 5). Furthermore, its role in caries prevention is recognized as one of the ten most significant public health achievements of the 20th century, with many studies indicating a substantial reduction in dental caries in populations consuming fluoridated water (6). The use of drinking water as a vehicle for fluoride (F) is internationally acknowledged, alongside milk and salt, as a highly cost-effective method (7), there by supporting its implementation as a community health measure.

Despite the various forms of fluoride delivery, the range of community engagement efforts, and some implementation challenges, Community Water Fluoridation (CWF) remains the most common and cost-effective method of delivering fluoride to communities (8). Studies indicate that communities with fluoridated water experience a 35% reduction in dental caries among children and a 26% reduction among adults (9). CWF has long been a public health strategy aimed at reducing dental caries, particularly among vulnerable populations.

CWF, the process of adjusting fluoride levels in public water supplies, has been a cornerstone of public health policy aimed at reducing dental caries and improving oral health across populations. However, disparities in access to fluoridated water exist, particularly in rural and underserved areas (10). While the use of community water supplies to provide fluorides is regarded as the most practical and effective measure for dental caries prevention (11, 12), the lack of water supply is not an insurmountable obstacle to receiving the benefits of fluorides. Several studies and public health programs have shown that alternative methods are effective in preventing dental caries (11, 13). When people do not have access to a public water supply, some countries have tested and implemented other community-based fluoridation programs at local, regional, or national levels.

Current fluoridation initiatives extend beyond water systems to include various alternative delivery methods aimed at enhancing oral health. Milk fluoridation programs have been implemented in countries where water fluoridation is not feasible, involving the addition of fluoride to milk distributed in schools and communities to provide children with a fluoride source (14, 15). Fluoride mouth rinses, which are particularly effective in school settings, and have been shown to reduce caries incidence, especially among populations with limited access to dental care (13). Other fluoride delivery methods, such as topical applications and fluoride varnishes, are commonly utilized in clinical settings for targeted caries prevention (16). However, they are also implemented as community-level interventions, where they have demonstrated substantial effectiveness in reducing caries incidence, particularly in high-risk populations, along with favourable cost-effectiveness profiles compared to other preventive strategies (17). Currently, silver diamine fluoride (SDF) has reemerged as a treatment for caries management, effectively arresting carious lesions in children (18). SDF also prevents root caries in older adults (19).

Additionally, there are regions in every country that have naturally occurring fluoride in their drinking water, with concentrations that fluctuate across a broad spectrum—from very low levels (ranging from 0.01 to 0.30 mg/L) to above the optimal level (20).

From a public health point of view, CWF is regarded as the most effective method of preventing dental caries in children. More than 500 million people worldwide use CWF (21, 22). However, despite this evidence-based support, barriers including political, administrative, financial, geographic, and technical factors have inhibited the achievement of this benefit for a large part of the world's population, especially in third-world countries and in non-metropolitan areas of developed countries.

Chile has a long history of fluoridation strategies. In Chile, the adoption and expansion of fluoridation initiatives reflect a broader commitment to improving oral health outcomes. CWF was carried out on a small scale in the cities of San Fernando and Curico in 1953 and was implemented nationally in the 1960s. Since then, despite some interruptions during the 1970s (23), CWF has been one of the cornerstone strategies for the prevention and control of dental caries in Chile. Unlike other countries in Europe, Latin America and the Caribbean, the use of salt as a vehicle for fluoride has never been considered by the Chilean Ministry of Health (21, 24, 25).

This manuscript explores the different fluoridation programs implemented in Chile, beginning with a historical overview of water fluoridation practices, including key milestones and governmental support for their expansion. The manuscript begins with an examination of the current oral health profile in the country, then examines current fluoridation initiatives, analyzing various delivery methods such as water and milk fluoridation, detailing the adoption of water fluoridation and exploring the associated challenges and controversies. The importance of community engagement efforts and public perceptions is also highlighted, with attention to challenges such as misinformation and regulatory inconsistencies that can impact program effectiveness. The paper concludes with a discussion of future directions for fluoridation initiatives, outlining potential strategies and considerations for improving oral health outcomes in Chile, emphasizing the potential of new technologies and alignment with the United Nations Sustainable Development Goals (SDGs) to help illustrate how good oral health can influence health outcomes (26).

The manuscript contributes to the existing literature by improving the understanding of fluoridation programs in Chile, a country where oral health disparities persist despite various public health initiatives. By examining diverse delivery methods and emphasizing the need for community engagement, the review highlights the complexities of implementing effective oral health strategies. The review aims to provide valuable insights for policymakers and public health practitioners by offering a comprehensive overview of fluoridation's historical significance, current practices, and future possibilities, contributing to public health policy and enhancing community health and well-being.

## 2 Oral health profile in Chile

Oral health is a national priority in Chile, recognized as fundamental for the well-being of individuals and communities (27). As in many countries, over the last few years there has been a significant decline in the prevalence of dental caries among children (28). Still, oral diseases and conditions are the most prevalent health conditions and represent a serious public health issue due to their impact on quality of life, and high treatment costs (29, 30). Despite significant improvements, dental caries remains a significant health problem; 50% of 4-year-olds have a history of dental caries, and more than half (55%) of individuals aged 15 years and older present non-treated carious lesions (31–33). Since 2000, oral health has been integrated into Chile's Health Objectives of the Decade, prioritizing caries prevention in individuals under 20 years of age (33).

The main causes of dental caries in Chile, as in many countries, are the consumption of sugar in food and drinks, a lack of exposure to the preventive effects of fluoride and plaque on teeth (34). These causes can be addressed through preventive measures such as exposure to fluoride treatments and sugar taxes (35). These two public health measures are specifically designed to reduce the health risks linked to the consumption of sugary products and to enhance dental health. These initiatives reflect the growing recognition of the need for proactive strategies to promote public health and mitigate the long-term costs associated with diet-related diseases and dental problems. Sugar taxes have been implemented in 31 countries, with many others at different stages of developing policy measures aiming to reduce free sugars intake (35). Sugar taxes are part of a comprehensive approach to addressing public health challenges related to excessive sugar consumption. In fact, the use of sugar taxes as a health promotion measure fits well within the principles of the Ottawa Charter for Health Promotion, through creating supportive environments (36).

Nonetheless, it is important to acknowledge that dental caries is multifaceted and influenced by a variety of factors beyond exposure to fluorides and sugar consumption. Dental caries continues to exist in fluoridated communities and in countries where a sugar tax has been implemented (37). Among other factors influencing the prevalence of caries are the social determinants of health, which encompass the conditions in which individuals are born, live, work, and age (38). By considering these determinants, public health initiatives can work towards more comprehensive strategies that address not only dietary habits but also the underlying factors that contribute to health disparities within communities.

Chile's public oral-health system is organized around the GES (Explicit Health Guarantees) Comprehensive Oral-Health guarantee for 6-year-old girls and boys, which requires health networks to provide a full dental check-up, preventive education and fluoride-based protection for every child at the start of primary school. This guarantee is intended to reduce the country's persistent caries burden by ensuring that all children receive timely, equity-focused services (39, 40).

One of the main GES-linked interventions is the “Sembrando Sonrisas” program, a complex preventive package for preschoolers (2–5 years) that includes supervised daily tooth-brushing with fluoride toothpaste, distribution of toothbrushes and twice-yearly applications of 5% sodium fluoride varnish in nurseries and schools (41).

In rural and semi-urban areas where CWF is limited or nonexistent, Chile has complemented GES with a fluoridated-milk program delivered through the national School Feeding Program (PAE). Over 35 000 children receive milk fortified with 0.65 mg fluoride per day, producing a 24%–27 % reduction in DMFT among 9- and 12-year-olds after 36 months (42).Together, these GES-mandated services—regular examinations, fluoride varnish, fluoride-toothpaste programs and fluoridated milk—form a coordinated, evidence-based approach that targets the two primary drivers of childhood caries: high sugar consumption and insufficient exposure to preventive fluoride. Both of the two primary drivers of childhood caries are shaped by broader social determinants such as socioeconomic status, geographic access to water fluoridation and educational resources. The GES program includes a range of oral health guarantees aimed at various population groups. It includes the GES Outpatient Dental Emergency Services, which provide urgent treatment for anyone in need regardless of ability to pay and the GES Comprehensive Oral Health Care for Pregnant Women, comprised of preventive visits, dental cleanings, and treatment for any conditions that may impact maternal or fetal health.

For adults aged 60 and over, the GES Comprehensive Dental Care for Adults ≥ 60 Years provides full-service dental care encompassing preventive, restorative, and prosthetic procedures designed to maintain oral function and enhance the quality of life for older adults (43).

Other GES-linked interventions include:

- Comprehensive Dental Care Program for last year High School Students—an integrated dental-care program for students in the fourth year of secondary school, providing regular check-ups, oral hygiene education, and necessary treatments to ensure healthy teeth and gums before they enter higher education or the workforce (44).
- The CERO (from Spanish: Control con Enfoque de Riesgo Odontológico (Focus on Oral Risk Control) program is a Chilean public-health initiative that shifts dental care from a purely curative model to a preventive, risk-based approach. Implemented in primary-care centers (CESFAMs), the program targets children—especially those in preschool and early school age—by conducting a systematic oral-risk assessment and delivering individualized preventive actions such as supervised tooth-brushing, fluoride-varnish applications, sealants, and tailored oral-hygiene education (45). By focusing on early detection and individualized prevention strategies, CERO aims to reduce the prevalence and severity of dental caries, particularly among socioeconomically disadvantaged families, and to promote long-term oral-health equity. An evaluation of the CERO program found balanced gender participation (51 % male, 49 % female) and a high proportion of children aged 6 years (25 %) and 4 years (≈20 %), indicating good reach within the target age groups and supporting the program's potential for broader application within Chile's public-health system (46).

## 3 Fluoridation programs in Chile

### 3.1 Adoption of water fluoridation in Chile

In Chile, as in many other countries, the fluoridation of drinking water is the main strategy for the prevention and control of dental caries (27). The origins of water fluoridation in Chile can be traced back to the mid-20th century. The initial adoption was influenced by emerging scientific evidence from the United States and Europe that demonstrated the efficacy of fluoride in preventing dental decay. The initial CWF intervention began in 1953 and resulted in a substantial decrease in dental caries in the cities where it was implemented (47). The city of Curico became the first in Chile to implement CWF (48), marking a significant milestone in the nation's public health policy. This early adoption laid the groundwork for subsequent initiatives to enhance oral health across the country. The decision was informed by studies indicating a substantial reduction in caries rates among children exposed to fluoridated water.

Following the successful implementation of water fluoridation in Curico, the Chilean government recognized the need for broader fluoridation efforts. Throughout the 1960s and 1970s, a series of key milestones facilitated the expansion of fluoridation programs. Government support was instrumental in promoting fluoridation as a public health priority. In 1964, a national health policy was established advocating for the fluoridation of drinking water to reduce the prevalence of dental diseases. The expansion of these programs was further supported by international partnerships and funding from organizations such as the World Health Organization (WHO) and the Pan American Health Organization (PAHO). These collaborations provided technical assistance and resources necessary for the implementation and maintenance of fluoridation systems, thereby enhancing their reach and effectiveness.

The earliest national regulations for water fluoridation were from 1969 (Decreto Supremo N° 735 del 7/11/1969). However, while its constitutionality was clearly established, the national program was interrupted in 1976 (23), and by the mid-1980s, the prevalence of caries had risen to critical levels, comparable to that in countries lacking fluoridation programs (49). In July 1981, a new legislative effort was launched with the creation of the “National Program for the Fluoridation of Drinking Water Supplies” (Decreto Supremo No. 915 con fecha 8/07/1981).

In 1985, the first water fluoridation program under this new fluoridation legislation began in the Valparaíso Region. Since then, the program has expanded, and currently approximately 82.3% of the Chilean population accesses water systems whose fluoride concentration has been adjusted to optimal levels for the prevention of dental caries (50), reflecting a growing consensus on the importance of fluoride in public health.

Today, Chile has a comprehensive range of fluoridation initiatives aimed at promoting oral health among its population (33). These initiatives include CWF, which is the primary method of fluoride delivery, ensuring that a significant portion of the population benefits from reduced caries rates (23). In addition to CWF, fluoride programs include:

- **Fluoridated Milk**: Recognizing the importance of milk as a dietary staple, particularly for children, Chile has introduced fluoridated milk programs. This initiative aims to supplement fluoride intake among children, especially in areas where water fluoridation may not be feasible (27).

While In Chile, CWF has been the main strategy for preventing caries, due to certain technical, geographic, administrative, and political circumstances prevalent in the country, the benefits of water fluoridation have not yet reached 100 per cent of the population. Populations living in rural areas, in particular small rural localities, have virtually no way of accessing continuous fluoride exposure, beyond the use of fluoridated toothpaste at home. To address this lack of access to water fluoridation, alternative fluoridation methods, such as fluoridated milk, are being explored (14).

The value of milk as an alternative vehicle for the administration of fluorides for caries prevention in humans has been reported since the 1950s (14). The world's first community project for milk fluoridation was established in Bulgaria in 1988 (7). Apart from Chile, community demonstrations are planned or conducted in China, Russia, Thailand, and the United Kingdom (7).

Fluoridated milk can be produced in liquid and powder forms. Several fluoride compounds can be used, including calcium fluoride, sodium fluoride (NaF) and disodium monofluorophosphate (MFP) (World Health Organization, 2005). In the 1990s milk fluoridation schemes in Chile utilized powdered milk fluoride with disodium monofluorophosphate (MFP) to enhance oral health, particularly in economically deprived areas with high caries. These began as community trials (42, 51). Most fluoridated milk programs use liquid milk and NaF as the fluoride compound. Powdered milk and MFP are used in the Chilean milk scheme (42, 51).

The Chilean government, through the National Board of Students Assistance and Scholarships (JUNAEB) implements programs to assist the country's most vulnerable school students. Within this context, JUNAEB's oral health program gives priority to the prevention of dental caries, through the implementation of programs aimed at both the individual and community levels. One such dental caries prevention program is the addition of fluorides to the school food program (PAE-F); fluoridated milk is distributed as part of breakfasts delivered every day by JUNAEB to children from rural schools who do not have access to water fluoridation. Students are eligible for this benefit if they are enrolled in a rural school without CWF, located in the regions of Coquimbo, Valparaíso, Metropolitan, O'Higgins, Maule, Ñuble, Biobío, La Araucanía, Los Ríos, Los Lagos, Aysén, and Magallanes (52).

In this way, this program of fluoridated milk in PAE-F achieves equity of access to fluoride for school children at risk of dental caries. Today, about 35000 school students from Year 1 to Year 8 (about 6–14 years of age) participate in the PAE-F program, receiving fluoridated milk to promote dental health (52).

This initiative ensures that children living in rural communities receive essential fluoride for oral healthcare, which is particularly crucial during their formative years when they are most susceptible to cavities. However, the program is inherently a temporary measure, typically benefiting children only until they reach approximately 12–14 years of age, when they depart from primary education. After this age, other oral health programs are designed to support both secondary students and adults in maintaining their dental health.

Despite its limitations, the Milk-F program provides schoolchildren with a significant head start in both education and health, fostering better oral hygiene practices and enhancing their overall well-being as they transition into adolescence and beyond. By integrating nutritional support with preventive dental care, Milk-F not only addresses immediate health needs but also lays the groundwork for lifelong healthy habits.

- **Fluoride Varnish:** Fluoride varnish was incorporated into Chile's public-health strategy as part of the Sowing Smiles program in 2015. This program aims to maintain and improve the oral health of the preschool population in municipal and subsidized schools by promoting healthy habits and implementing targeted protective measures. The use of varnishes is reinforced within the regulations on the use of fluorides in dentistry, which emphasize that the application of a 5 % sodium-fluoride varnish by qualified dental personnel is recommended at least twice a year for preschool-aged children and for any child with early-stage carious lesions. Integration of varnish applications into school-based oral-health programs and routine pediatric dental visits has contributed to a measurable reduction in caries incidence in the Chilean paediatric population, complementing community-wide measures such as water, and milk fluoridation (27).
- **Fluoridated toothpaste:** Toothpaste containing fluoride is the cornerstone of individual caries-prevention strategies in Chile, as in most countries. In 2015, the Chilean Ministry of Health introduced significant changes to the regulations governing toothpaste concentration. In this regulation, they advised the families that children should begin brushing with a fluoride toothpaste (≥1,000 ppm F⁻) as soon as the first tooth erupts and continue this practice twice-daily throughout life. Supervised brushing until the age of 6 years is also advised to ensure adequate dosage and avoid excessive fluoride ingestion (27).
- **Fluoride Mouthwash/Gel**: School-based programs that provide fluoride mouthwash and gels have historically been introduced to improve oral health among children, especially in schools serving lower-socio-economic communities (27). However, Chile's current school-based oral-health initiatives no longer include a fluoride-mouthwash/gel component. The national “Sembrando Sonrisas” program now focuses on daily supervised tooth brushing with fluoride toothpaste and biannual fluoride-varnish applications, while commercial fluoride rinses remain available only as private products. The current fluoride regulation recommends prescribing mouthwashes and gels based on the patient's caries risk and using varnishes instead of gels in preschoolers (27).

**Figure 1:**
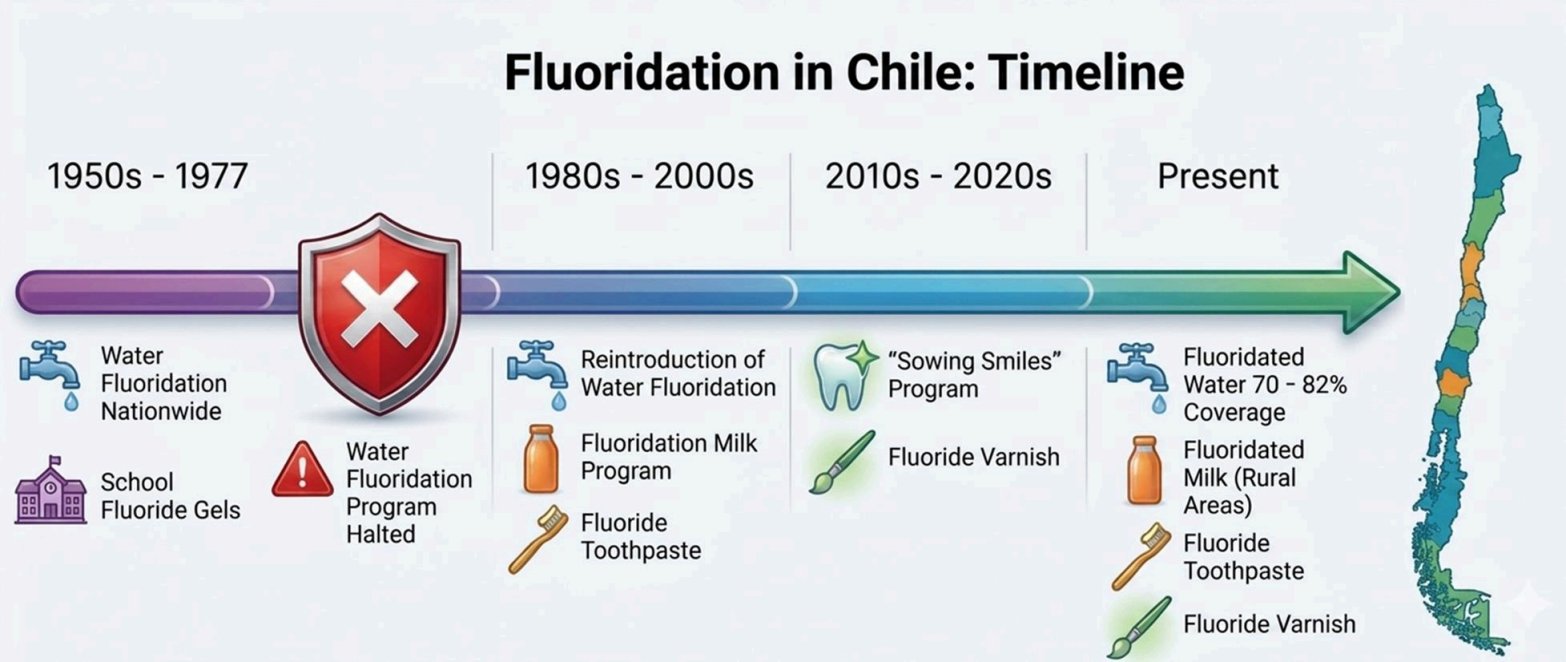
Fluoridation in Chile: timeline. References (23, 27).

**Figure 2:**
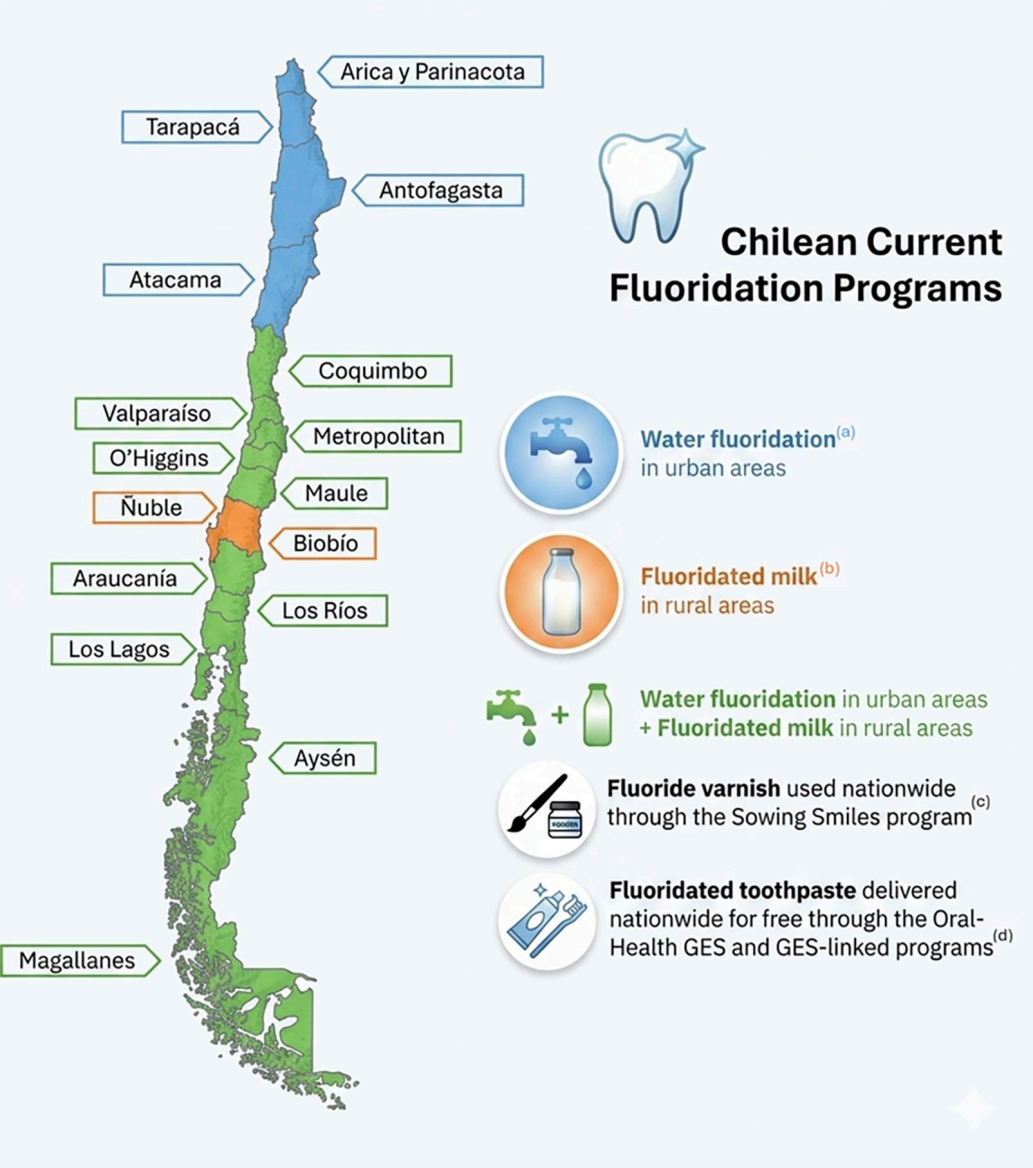
Chilean current fluoridation programs. References: **(a)** (68) **(b)** (52) **(c)** (41) **(d)** (40).

Additional programs that incorporate fluoridation include educational campaigns aimed at promoting good oral hygiene practices and regular dental check-ups during which the role of fluoride in maintaining oral health is emphasized (33). Figures 1,2 summarizes a timeline of tshe different fluoride programs implemented in Chile.

In Chile, the implementation of a sugar tax in 2014 marked a significant advance in public health policy and was aimed at combating rising obesity rates and related non-communicable diseases (53). This tax levies a fee on sugary beverages, with the aim of discouraging consumption and promoting healthier dietary choices. The revenue generated from the tax has been allocated to various health initiatives, including educational programs and subsidies for healthier food options. Following the introduction of the sugar tax, studies found that it had led to a reduction in the purchase of sugary drinks, demonstrating a positive shift in consumer behavior (53, 54).

### 3.2 Challenges and controversies

Despite the successes of fluoridation programs in Chile, including the groundbreaking sugar tax, several challenges and controversies persist (23). The effectiveness of these measures continues to be a topic of debate, as policymakers assess the long-term impacts on public health and the broader food environment in Chile, and grapple with pressure from the food and beverage industries (55).

#### 3.2.1 Public perception and misinformation

Public perception of fluoride can be a contentious issue, with a growing anti-fluoride movement gaining traction in various communities (16). Anti-fluoridation movements often cite concerns about potential health risks and conspiracy theories around who truly benefits, despite a lack of scientific evidence supporting these claims (16). Misinformation regarding the safety and efficacy of fluoride has fueled skepticism among segments of the population about potential health risks associated with fluoride consumption, despite the robust, long term, body of scientific evidence supporting its safety (16, 56). Misconceptions and conspiracy theories surrounding fluoride have proliferated, leading to resistance against fluoridation initiatives (16). This has necessitated a proactive approach by health authorities to address misconceptions and reinforce the evidence-based benefits of fluoridation.

Sexton et al. (57) investigated decisions made by local governments regarding the cessation or non-implementation of CWF. This study highlighted structural and political barriers that can impede equitable CWF implementation, despite it being an evidence-based, cost-effective public health intervention. The findings highlight the influence of political, economic, and community factors that can outweigh public health research-based evidence. These problematic influences can undermine the equity-focused intent of CWF, increasing health inequities. Such opposition to fluoridation reflects values-based arguments rather than factual evaluations, emphasizing the influence of misinformation and the challenges faced in science-based policymaking (57). To address these issues, the study calls for policy reforms that restore decision-making authority to well-resourced health authorities and ensure community engagement is complemented by expert guidance.

Furthermore, despite the safety of water fluoridation being repeatedly re-evaluated and confirmed and the safety of fluoride consumption having been comprehensively substantiated by several systematic reviews of the effectiveness of water fluoridation in preventing dental caries (6, 58, 59), the debate regarding the use of fluoride to prevent dental caries continues. Recently, Utah became the first U.S. state to ban fluoride in public water due to concerns raised by the U.S. health secretary about potential health risks. Signed into law by Governor Spencer Cox, the ban took effect on May 2025, and other states are considering similar legislation. CWF has faced criticism from experts who worry this move will negatively impact oral health (60). Regardless, the evidence in support of fluoridation continues, for example, Choi and Simmon (61) simulated the outcomes of discontinuing public water system fluoridation and anticipated a 7.5 percentage point increase in tooth decay among children, alongside estimated costs of around US$9.8 billion over a five-year period. This increase in dental expenses and resulting disproportionate health impacts would primarily affect both publicly insured and uninsured children.

As the above discussion demonstrates, continuous effective community engagement and public awareness campaigns are critical components of successful oral health programs. Educating the public about the benefits of fluoride in preventing dental caries is vital. Campaigns should focus on the importance of CWF and other fluoride programs, sugar consumption reduction, and regular dental check-ups. Public health campaigns aimed at informing communities about the benefits and safety of fluoridation have been shown to enhance acceptance and participation (62, 63). These activities are critical components of Chile's fluoridation initiatives (64, 65). Furthermore, public health campaigns are designed to inform communities about the benefits of fluoride and dispel common misconceptions. Educating healthcare providers and dental professionals about fluoride's role in oral health is also essential for fostering community support. Educational materials are distributed in schools, healthcare facilities, and community centres, emphasizing the importance of fluoride in preventing dental diseases. Moreover, community forums and workshops provide platforms for dialogue, allowing residents to express concerns and receive accurate information regarding fluoride use (66, 67).

#### 3.2.2 Regulatory and implementation challenges

Regulatory challenges can also hinder the implementation or expansion of fluoridation programs. Variability in local governance, local regulations and resource allocation can lead to inconsistencies in program implementation across regions, and therefore disparities in exposure. Additionally, funding constraints and political opposition can impede the establishment of new fluoridation initiatives, particularly in economically disadvantaged areas (10). Demonstrating the impact of the politicization of this important public health issue, in 2007, the Biobío Region of Chile opposed CWF, with various arguments (23). Later, in 2018, the Ñuble Region officially split from the Biobío Region, leaving both as the only regions in Chile without CWF or any form of systemic fluoridation (68, 69).

Economic Evaluation (EE) is an integral part of the planning and delivery process of health services and is defined as “the comparative analysis of alternative lines in terms of their costs and consequences to assist policy decisions” (70). There is a considerable volume of literature regarding the economic value of water fluoridation, and such research continues to support its use (8, 71). Economic evaluations of fluoridation programs have been conducted in Chile, specifically focused on CWF (72) and fluoridated milk (73). The CWF study concluded that an investment of RCH 140.22 (US$1.40) per child annually resulted in a net savings of RCH 8,930.49 (US$14.9) for each tooth with dental caries history avoided (72). Similar results are obtained when using fluoridated milk (73, 74). Another study assessed the cost-effectiveness of seven dental caries prevention programs for schoolchildren in Chile (water fluoridation, dental sealants) and school-based (milk fluoridation, fluoridated mouthrinses, APF-Gel, supervised tooth brushing) from a societal perspective, comparing intervention groups with non-intervention communities (74). To put these results in context, Salt-F was also included in the analysis, although there is no Salt-F operating in Chile. The analyses indicated that water, salt, and milk fluoridation, along with fluoride mouth rinses (FMR), were cost-effective programs that yield savings even under conservative assumptions. In contrast, supervised toothpaste use, dental sealant placement, and APF-Gel application incur costs to society, as their expenses exceed the costs of restorative treatments that would be needed if these preventive measures were not implemented.

Zaror and collaborators (75) evaluated the effectiveness, safety, and cost-effectiveness of oral health policies and programs implemented in Chile, such as water fluoridation, milk fluoridation and the “Sembrando Sonrisas” initiative (41). Importantly, the findings from this systematic review indicated a scarcity of robust evidence assessing the impact of public oral health programs in Chile. While many interventions showed positive outcomes, the low methodological quality of studies raises concerns regarding the reliability of these results. The interconnected nature of the programs further complicates the evaluation of individual interventions.

While many strategies have shown positive impacts on oral health, the evidence remains limited and methodologically weak, posing challenges for informed decision-making. Recommendations from Zaror and collaborators (75) include conducting thorough evaluations of programs from implementation, ensuring a focus on relevant outcomes for decision-makers and patients, and incorporating local effectiveness data for economic assessments. The study emphasized the need for future research to utilize robust methodologies that ensure the rational use of public resources, ultimately enhancing oral health and overall well-being in the population. The authors concluded that the limited evidence on the impact of oral health programs hinders informed decisions in Chile.

## 4 Future directions

Looking ahead, a clear trend in Chile is the ageing population. Currently, Chile is considered to be a country with “advanced ageing” and it is expected that by the year 2030 it will have the highest ageing index in the region, reaching a proportion of older people close to 28% by 2050, that is, approximately one in four people will be over 60 years of age (76). Parallel to this, there is also evidence of an epidemiological transition in oral health, moving from high rates of edentulism and tooth loss towards lower rates of edentulism and general improvement of oral health in the older population (77, 78). This creates new challenges to society and, more specifically, to the dental profession. For example, how to maintain the oral health of that group of the population. This confluence of public health trends raises the prospect that the cost-saving benefits of CWF might be offset to some degree by the potentially high costs of periodontal treatment needs later in life (79). The increased oral health needs of older Chileans, and the difficulties in providing access to proper oral health services in RACFs, will become a challenge for the entire society and, in particular, for oral health professionals. Fluoride is widely acknowledged as an effective protection against coronal caries in children. Its efficacy in older adults and in preventing root caries, however, is more limited (9).

In Chile, within the normative framework that regulates the health authorization of RACFs, the application of oral hygiene and fluoride exposure protocols and oral health aspects are not specifically contemplated (80), so oral care practices performed or supervised in the respective RACFs are unknown (81).

**Silver Diamine Fluoride (SDF)** has emerged as an innovative treatment option for managing dental caries, especially in young children and individuals with limited access to dental care. In Chile (SDF) is recognized as an effective and minimally invasive treatment for dental caries, particularly for early childhood caries and in managing older adults with sensitive teeth and/or root caries (82, 83).

While SDF is being integrated into the curriculum of Chilean dental schools, it is not included in a widespread national government program like “Sembrando Sonrisas”; which currently focuses on toothbrushing and fluoride varnish (84, 85). This suggests its use is primarily in private or academic settings. This effort will not only help to increase information about preventing and management root caries lesions of dentate older adults and in so doing, decrease disadvantages in oral health and help them maintain high levels of general health, quality of life, nutrition, and social interactions.

Given that SDF is an affordable, portable, and effective alternative to traditional restorative treatments, especially for underserved communities, its future use is likely to expand (86, 87). Global health policies are increasingly promoting SDF for caries control, and its cost-effectiveness makes it a strong candidate for inclusion in public health programs to improve oral health equity in Chile and other countries (86, 88).

The future of fluoridation programs in Chile will likely involve integrating new technologies and delivery methods, such as mobile health applications, to inform and educate communities and monitor fluoride levels (89). Additionally, continued investment in research and community education will be essential for dispelling myths and fostering trust in fluoridation initiatives. A multidisciplinary approach is needed to address existing challenges and enhance public acceptance of initiatives to improve dental public health through both preventive measures and economic incentives (e.g., fluorides and sugar tax). Furthermore, exploring alternative delivery methods, such as the integration of fluoride into dietary supplements or expanded access to dental care, may enhance the effectiveness of public health strategies. Collaborative efforts with international health organizations can also provide valuable insights and resources for optimizing fluoridation programs.

## 5 Final remarks

Fluoridation is a vital and proven public health strategy for preventing dental caries and promoting oral health. Chile's history of fluoridation illustrates a commitment to evidence-based public health initiatives. Proper management of fluoride exposure is essential for achieving optimal oral health outcomes. Furthermore, among all forms of fluoridation, CWF has been found to be the most cost-effective way for lowering the risk of caries in low-income, disadvantaged communities (90). This is consistent with the concept of “Leaving no one behind” in the United Nations Sustainable Development Goals (SDGs) (91, 92).

Furthermore, fluoride has been repeatedly term, demonstrated to reduce oral health problems and improve overall health outcomes and quality of life (SDG 3: Good Health and Well-being). Additionally, addressing dental caries, particularly in children, supports SDG 4 (Quality Education) by enhancing their ability to learn and thrive. To combat oral health disparities related to socioeconomic barriers, Oral Health Practitioners (OHPs) should advocate for government support and expanded access to oral healthcare, especially through fluoridation programs that assist disadvantaged populations, linking to SDG 1 (No Poverty) and SDG 10 (Reduced Inequalities). Finally, addressing discrimination based on social determinants of health is critical for access to good oral health care, underscoring the obligation of OHPs to champion policies that promote equity, education, and social inclusion, thereby fostering overall health and well-being in line with SDG 5 (Gender Equality). Furthermore, ensuring safe drinking water free from harmful contaminants aligns with SDG 6 (Clean Water and Sanitation), as sustainable fluoride practices support clean water initiatives and ecological health.

While significant progress has been made in expanding fluoridation initiatives. There is much more that needs to be done to improve oral health in Chile. Ongoing challenges related to public perception and implementation must be addressed. A comprehensive approach that prioritizes community engagement, education, and regulatory support will be crucial for the continued success of fluoridation programs in Chile. By fostering an environment that prioritizes preventive oral health measures and equitable access to fluorides, Chile and similar nations, can enhance their oral health policies and significantly reduce the prevalence of dental caries among their populations. As the nation navigates the complexities of public health policy, the lessons learned from the fluoridation experience can serve as a valuable framework for future health initiatives aimed at enhancing oral health and overall well-being. Future efforts should focus on enhancing community engagement, collaboration between government health agencies, dental professionals, and educational institutions, leveraging new technologies, and continuing to disseminate evidence-based information at all levels to combat misinformation and politicization surrounding fluoride.

## References

[1] National Institute of Dental Research (NIDR). Fluoride in Drinking Water: A Review of the Evidence. Rockville, MD: National Institute of Dental Research (1990).

[2] National Institute of Dental and Craniofacial Research. The Story of Fluoridation. Bethesda, MD: NIDCR (2024). Available online at: https://www.nidcr.nih.gov/health-info/fluoride/the-story-of-fluoridation (Accessed March 3, 2026).

[3] Centers for Disease Control and Prevention. Community Water Fluoridation. 2022 Water Fluoridation Statistics. Atlanta, GA: CDC (2024). Available online at: https://www.cdc.gov/fluoridation/php/statistics/2022-water-fluoridation-statistics.html (Accessed March 3, 2026).

[4] World Health Organization. Follow up to the Political Declaration of the Third High Level Meeting of the General Assembly on the Prevention and Control of Non Communicable Diseases. Annex 3. Draft Global Strategy on Oral Health. Seventy fifth World Health Assembly (2022). Report No.: A75/10 Add.1. Available online at: https://apps.who.int/gb/ebwha/pdf_files/WHA75/A75_10Add1-en.pdf (Accessed March 3, 2026).

[5] World Health Organization. Global Status Report on Oral Health 2022. Geneva: WHO (2022). Available online at: https://www.who.int/team/noncommunicable-diseases/global-status-report-on-oral-health-2022 (Accessed March 3, 2026).

[6] Centers for Disease Control and Prevention. Fluoridation of public drinking water to prevent dental caries. Morb Mortal Wkly Rep. (1999) 48:933–90.

[7] Banoczy J Petersen PE Rugg-Gunn AJ. Milk Fluoridation for the Prevention of Dental Caries. Geneva: World Health Organization (2009).

[8] Mariño R Zaror C. Economic evaluations in water-fluoridation: a scoping review. BMC Oral Health. (2020) 20(1):115. 10.1186/s12903-020-01100-y 32299417 PMC7164347

[9] Iheozor-Ejiofor Z Worthington HV Walsh T O'Malley L Clarkson JE Macey R Water fluoridation for the prevention of dental caries. Cochrane Database Syst Rev. (2015) 2015(6):CD010856. 10.1002/14651858.CD010856.pub2 Update in: Cochrane Database Syst Rev. 2024 October 4;10:CD010856. 26092033 PMC6953324

[10] Burt BA Eklund SA. Dentistry, Dental Practice, and the Community. St Louis: Elsevier (2005).

[11] Centers for Disease Control and Prevention. Recommendations for using fluoride to prevent and control dental caries in the United States. Morb Mortal Wkly Rep. (2001) 50(RR14):1–42. 11521913

[12] Horowitz HS. Decision-making for national programs of community fluoride use. Community Dent Oral Epidemiol. (2000) 28:321–9. 10.1034/j.1600-0528.2000.028005321.x 11014508

[13] NHS England/Department of Health and Social Care. Delivering Better Oral Health: An Evidence Based Toolkit for Prevention. 2025. Chapter 9: fluoride. Available online at: http://GOV.UK (Accessed March 3, 2026).

[14] Mariño R. Should we use milk fluoridation? A review. Bull Pan Am Health Organ. (1995) 29:287–98. 8605520

[15] Yeung CA Chong LY Glenny AM. Fluoridated milk for preventing dental caries. Cochrane Database Syst Rev. (2015) 2015(9):CD003876. 10.1002/14651858.CD003876.pub4 26334643 PMC6494533

[16] Samaranayake L Porntaveetus T Tsoi J Tuygunov N. Facts and fallacies of the fluoride controversy: a contemporary perspective. Int Dent J. (2025) 75(4):100833. 10.1016/j.identj.2025.04.013 40359684 PMC12141933

[17] Zaror C Muñoz-Millán P Espinoza-Espinoza G Vergara-González C Martínez-Zapata MJ. Cost-effectiveness of adding fluoride varnish to a preventive protocol for early childhood caries in rural children with no access to fluoridated drinking water. J Dent. (2020) 98:103374. 10.1016/j.jdent.2020.103374 32413383

[18] Crystal YO Marghalani AA Ureles SD Wright JT Sulyanto R Divaris K Use of silver diamine fluoride for dental caries management in children and adolescents, including those with special health care needs. Pediatr Dent. (2017) 39(5):135–45. 29070149

[19] Grandjean ML Maccarone NR McKenna G Müller F Srinivasan M. Silver diamine fluoride (SDF) in the management of root caries in elders: a systematic review and meta-analysis. Swiss Dent J. (2021) 131(5):417–24. 10.61872/sdj-2021-05-02 33515230

[20] Browne D Whelton H O’Mullane D. Fluoride metabolism and fluorosis. J Dent. (2005) 33:177–86. 10.1016/j.jdent.2004.10.003 15725518

[21] O'Mullane DM Baez RJ Jones S Lennon MA Petersen PE Rugg-Gunn AJ Fluoride and oral health. Community Dent Health. (2016) 33(2):69–99. 27352462

[22] World Health Organization. The World Oral Health Report 2003. Geneva: WHO (2003). Available online at: http://www.who.int/oral_health (Accessed March 3, 2026).

[23] Biblioteca del Congreso Nacional de Chile. Cronología de la fluoruración de agua potable en Chile y situación de la región del Biobío (2018). Available online at: https://obtienearchivo.bcn.cl/obtienearchivo?id=repositorio%2F10221%2F24971%2F2%2FInforme_Fluoracion_Agua_Potable.pdf (Accessed March 3, 2026).

[24] Estupiñan S. Promoting Oral Health: The Use of Salt Fluoridation to Prevent Dental Caries. Washington, DC: Pan American Health Organization (2005).

[25] Marthaler TM Menghini G Steiner M Sener-Zanola de Crousaz P Corti M Eckardt A. Excreción urinaria de fluoruro en niños que consumen suplementos de fluoruro en la sal o el agua. Arch Odontoestomatol Prev Comunitaria. (1992) 4:27–35.

[26] Watt RG. From victim blaming to upstream action: tackling the social determinants of oral health inequalities. Community Dent Oral Epidemiol. (2007) 35(1):1–11. 10.1111/j.1600-0528.2007.00348.x 17244132

[27] Ministerio de Salud de Chile. Normas de Uso de Fluoruros en Odontología. Santiago: Departamento Salud Bucal, División de Prevención y Control de Enfermedades, Subsecretaría de Salud Pública. Ministerio de Salud de Chile (2025).

[28] Cleaton-Jones P Fatti P Bonecker M. Dental caries trends in 5- to 6-year-old and 11- to 13-year-old children in three UNICEF designated regions: Sub Saharan Africa, Middle East and North Africa, Latin America and Caribbean: 1970–2004. Int Dent J. (2006) 56:294–300. 10.1111/j.1875-595X.2006.tb00104.x 17069073

[29] Bernabe E Marcenes W Abdulkader RS Abreu LG Afzal S Alhalaiqa FN Trends in the global, regional, and national burden of oral conditions from 1990 to 2021: a systematic analysis for the global burden of disease study 2021. Lancet. (2025) 405(10482):897–910. 10.1016/S0140-6736(24)02811-3 40024264

[30] Peres MA Macpherson LMD Weyant RJ Daly B Venturelli R Mathur MR Oral diseases: a global public health challenge. Lancet. (2019) 394(10194):249–60. 10.1016/S0140-6736(19)31146-8.6736(19)32079-3 31327369

[31] Ministerio de Salud. Informe Consolidado: Diagnóstico Nacional de Salud Bucal de los Niños y Niñas de 2 y 4 Años que Participan en la Educación Parvularia. Chile 2007–2010. Santiago, Chile: Ministerio de Salud (2015c. Available online at: https://diprece.minsal.cl/wrdprss_minsal/wp-content/uploads/2015/05/Informeconsolidado-2-y-4-a%C3%B1os.pdf (Accessed May 08, 2026).

[32] Ministerio de Salud. Encuesta Nacional de Salud. Informe Salud Bucal. Chile 2016–2017. Santiago, Chile: Ministerio de Salud (2019). Available online at: http://epi.minsal.cl/wp-content/uploads/2021/03/Informe_Salud_Bucal_ENS_2016_17.pdf (Accessed May 08, 2026).

[33] Ministerio de Salud. Plan Nacional de Salud Bucal 2021–2030. Santiago, Chile: Ministerio De Salud (2021). Available online at: https://diprece.minsal.cl/wp-content/uploads/2022/01/PLAN-NACIONAL-DE-SALUD-BUCAL-2021-2030.pdf (Accessed May 08, 2026).

[34] Victoria State Government, Department of Health and Human Services. Victorian Action Plan to Prevent Oral Disease 2020–30 (2020). Available online at: https://www.ohv.org.au/__data/assets/pdf_file/0016/250900/victorian-action-plan-to-prevent-oral-disease-2020.pdf (Accessed May 08, 2026).

[35] World Health Organization. Sugars and Dental Caries (2025b). Available online at: https://iris.who.int/server/api/core/bitstreams/3a21b73f-c388-4bc0-8ac6-c16f334dc0d9/content (Accessed March 3, 2026).

[36] World Health Organization. Ottawa Charter for Health Promotion (1986). Available online at: https://www.canada.ca/content/dam/phac-aspc/documents/services/health-promotion/population-health/ottawa-charter-health-promotion-international-conference-on-health-promotion/charter.pdf (Accessed April 13, 2026).

[37] Cockcroft B. Water fluoridation, myths, politics and evidence. Br Dent J. (2025) 238:245. 10.1038/s41415-025-8411-2 40021873

[38] World Health Organization. Social Determinants of Health (2025). Available online at: https://www.who.int/health-topics/social-determinants-of-health#tab=tab_1 (Accessed May 08, 2026).

[39] Celis A Conway DI Macpherson LMD McMahon AD. Data resource profile: national child oral health improvement programmes for Chile. Int J Epidemiol. (2023) 52(2):e110–5. 10.1093/ije/dyac191 36264249 PMC10115402

[40] Ministerio de Salud. GPC Salud Oral Integral para niños y niñas de 6 años (2013). ISBN 978 956 8823 11 5. Available online at: https://diprece.minsal.cl/wrdprss_minsal/wp-content/uploads/2014/12/Salud-Oral-Integral-ni%C3%B1os-y-ni%C3%B1as-6-a%C3%B1os.pdf (Accessed March 3, 2026).

[41] Ministerio de Salud. Orientaciones Técnico Administrativas Para La Ejecución Del Programa Sembrando Sonrisas 2020 (2020). Available online at: https://www.sscoquimbo.cl/gob-cl/procesos/files/21-04-2020/INFORMACION%20REFERENTE%20ODONTOLOGICO/OT%20PRAPS%20ODONTOLOGICOS%202020/Orientacion%20Tecnica%20Programa%20Sembrando%20Sonrisas%202020.pdf (Accessed March 3, 2026).

[42] Weitz A Mariñanco MI Villa A. Reduction of caries in rural school-children exposed to fluoride through a milk-fluoridation programme in araucania, Chile. Community Dent Health. (2007) 24(3):186–91. 17958081

[43] Ministerio de Salud. Problemas de Salud—AUGE. Santiago: Ministerio de Salud, Chile (2026). (Accessed March 3, 2026).

[44] Ministerio de Salud (Chile). AtenciÃ³n OdontolÃ³gica. Santiago: DIPRECE. Available online at: https://diprece.minsal.cl/programas-de-salud/salud-bucal/informacion-a-la-comunidad-salud-bucal/atencion-odontologica/ (Accessed May 8, 2026).

[45] Ministerio de Salud (Chile). Orientación Técnico Administrativa Población en Control con Enfoque de Riesgo Odontológico: Programa CERO. Santiago, Chile: Ministerio de Salud (2019). Available online at: https://diprece.minsal.cl/wp-content/uploads/2019/02/Orientacion-Tecnica-Programa-CERO-2019.pdf (Accessed May 08, 2026).

[46] Muñoz O Araneda J. Evaluation of the zero program in a cesfam in the south of Chile. Int J Odontostomat. (2022) 16(2):273–8. 10.4067/S0718-381X2022000200273

[47] Barros L Jiménez J Risnik A. Report on dental caries and other oral diseases in schoolchildren from Curico and san fernando. Revista de Medicina Preventiva y Social del SNS. (1964) 4:3–4.

[48] Adriasola G. First evaluation of the program of fluoridation of drinking water in Curico-San Fernando, Chile, 1956. Bol Oficina Sanit Panam. (1959) 47:412–20. 13791948

[49] Urbina T Vincent M Nacrur JP Vargas S. Dental caries in pre-school and basic school children from Santiago. Rev Dental Chile. (1987) 18:43–58.

[50] Gómez S Fernández O. Fluoración del agua potable: experiencia en Chile. In: Gómez S, editor. Fluorterapia en Odontología Para Niños y Adultos. 4th ed. Valparaiso: Santiago Gomez Soler (2010). p. 211–21.

[51] Mariño R Villa AE Guerrero S. A community trial for fluoridated milk in Chile. Community Dent Oral Epidemiol. (2001) 29:435–42. 10.1034/j.1600-0528.2001.290604.x 11784286

[52] Junta Nacional de Auxilio Escolar y Becas (JUNAEB). Programa de Salud Oral (2026). Available online at: https://www.junaeb.cl/salud-oral/ (Accessed May 08, 2026).

[53] Cuadrado C Dunstan J Silva-Illanes N Mirelman AJ Nakamura R Suhrcke M. Effects of a sugar-sweetened beverage tax on prices and affordability of soft drinks in Chile: a time series analysis. Soc Sci Med. (2020) 245:112708. 10.1016/j.socscimed.2019.112708 31862547 PMC7267770

[54] Caro JC Ng SW Taillie LS Popkin BM. Designing a tax to discourage unhealthy food and beverage purchases: the case of Chile. Food Policy. (2017) 71:86–100. 10.1016/j.foodpol.2017.08.001 29375180 PMC5783649

[55] Fooks GJ Williams S Box G Sacks G. Corporations’ use and misuse of evidence to influence health policy: a case study of sugar-sweetened beverage taxation. Global Health. (2019) 15(1):56. 10.1186/s12992-019-0495-5 31551086 PMC6760066

[56] Anderson O. Community Water fluoridation: Untangling Facts from Fear. ADANews (June 30, 2025). Available online at: https://adanews.ada.org/ada-news/2025/june/community-water-fluoridation-untangling-facts-from-fear/5 (Accessed March 3, 2026).

[57] Sexton CT Barzi F Do L Lalloo R Stormon N. Government decisions to remove water fluoridation: a qualitative analysis. Crit Public Health. (2026) 36(1):2605755. 10.1080/09581596.2025.2605755

[58] Boehmer TJ Lesaja S Espinoza L Ladva CN. Community water fluoridation levels to promote effectiveness and safety in oral health—united States, 2016–2021. MMWR Morb Mortal Wkly Rep. (2023) 72(22):593–6. 10.15585/mmwr.mm7222a1 37261997 PMC10243485

[59] National Health and Medical Reserch Council. NHMRC Public Statement 2017: Water Fluoridation and Human Health in Australia. Canberra: NHMRC (2017).

[60] Yousif N. Utah Becomes First US State to Ban Fluoride in its Water. BBC News (March 30, 2025). Available online at: https://www.bbc.com/news/articles/c4gmggp2y99o (Accessed March 3, 2026).

[61] Choi SE Simon L. Projected outcomes of removing fluoride from US public water systems. JAMA Health Forum. (2025) 6(5):e251166. 10.1001/jamahealthforum.2025.1166. Erratum in: JAMA Health Forum. 2025 July 3;6(7):e252898. doi: 10.1001/jamahealthforum.2025.2898. 40445598 PMC12125645

[62] Lucas J McGregor RM Kiss S Perrella AML. Where public health meets public opinion: understanding political support for fluoridation in calgary, 2021. Can J Public Health. (2024) 116(2):309–15. 10.17269/s41997-024-00960-z 39557770 PMC12076985

[63] Yeung CA. Public opinion on community water fluoridation in Scotland. Br Dent J. (2024) 237:748. 10.1038/s41415-024-8114-0 39572794

[64] Ministerio de Salud (Chile). OrientaciÃ³n tÃ©cnica para el logro de las metas de salud bucal - ENS 2011-2020. Santiago: SubsecretarÃŁa de Salud PÃºblica, DIPRECE (2013). Available online at: https://diprece.minsal.cl/wrdprss_minsal/wp-content/uploads/2016/02/Orientaci%C3%B3n-t%C3%A9cnica-para-el-logro-de-las-metas-de-salud-bucal-ENS-2011-2020.pdf (Accessed May 8, 2026).

[65] Ministerio de Salud (Chile). OT Modelo Dirigido a Equipos Mesas Regionales 2017–2020. Santiago: Ministerio de Salud, Chile (2017). Available online at: https://diprece.minsal.cl/wp-content/uploads/2017/09/OT-Modelo-dirigido-a-Equipos-Mesas-Regionales-2017-2020.pdf (Accessed March 3, 2026).

[66] Kemp D Mackert M Bouchacourt L Lazard AJ Wolfe JO Stewart B Promoting support for community water fluoridation: testing message effects and the role of normative beliefs. J Am Dent Assoc. (2021) 152:1012–9. 10.1016/j.adaj.2021.06.002 34489066

[67] Mackert M Bouchacourt L Lazard A Wilcox GB Kemp D Kahlor LA Social media conversations about community water fluoridation: formative research to guide health communication. J Public Health Dent. (2021) 81:162–6. 10.1111/jphd.12404 33058200

[68] Ministerio de Salud de Chile. Cobertura de Fluoración del Agua Potable 2022. Santiago: Subsecretaría de Salud Pública (2022). Available online at: https://diprece.minsal.cl/wp-content/uploads/2022/11/COBERTURA-FLUORACION-AGUA-POTABLE-2022_nov-2022_-FINAL-VB.pdf (Accessed March 3, 2026).

[69] Ministerio de Salud de Chile. Informe Nacional de Flúor en Agua Potable por Regiones año 2023 con Datos 2022. Santiago: Ministerio de Salud, Chile (2023). Available online at: https://diprece.minsal.cl/wp-content/uploads/2024/04/2023.09.26_INFORME-FLUOR-AGUA-POTABLE-2023.pdf (Accessed March 3, 2026).

[70] Drummond M Sculpher M Torrance G O’Brien B Stoddart G. Methods for the Economic Evaluation of Health Care Programmes. 3^rd^ ed Oxford: Oxford University Press (2005).

[71] Ran T Chattopadhyay SK. Community preventive services task force. Economic evaluation of community water fluoridation: a community guide systematic review. Am J Prev Med. (2016) 50(6):790–6. 10.1016/j.amepre.2015.10.014 26776927 PMC6171335

[72] Mariño R Fajardo J Morgan M. Evaluación económica del programa de fluoración del agua de beber en Chile (economic evaluation of the water fluoridation program in Chile). Revista Chilena de Salud Pública. (2013) 17:124–31. 10.5354/0719-5281.2013.27092

[73] Mariño R Fajardo J Morgan M. Economic evaluation of dental caries prevention programs using milk and its products as the vehicle for fluorides: cost versus benefits. In: Watson R Gerald J Preedy V, editors. Dietary Supplements and Nutraceuticals: Cost Analysis Versus Clinical Benefits. New York: Springer: Humana Press (2011). p. 143–61.

[74] Mariño R Fajardo J Morgan M. Cost-effectiveness models for dental caries prevention programs among Chilean schoolchildren. Community Dent Health. (2012) 29:302–8. 23488214

[75] Zaror C Muñoz K Jans A Espinoza-Espinoza G. ¿Qué sabemos sobre el impacto de las políticas y programas ministeriales de salud oral en Chile?: revisión de alcance. Int J Interdiscip Dent. (2025) 18(1):47–54. 10.4067/s2452-55882025000100047

[76] Instituto Nacional de Estadísticas (Chile). Síntesis de Resultados Censo 2024. Santiago: Instituto Nacional de EstadÃŁsticas (Chile) (2025). Available online at: https://censo2024.ine.gob.cl/wp-content/uploads/2025/12/sintesis_resultados_censo2024.pdf (Accessed March 3, 2026).

[77] Beltrán V Flores M Sanzana C Muñoz-Sepúlveda F Alvarado E Venegas B Tooth loss and caries experience of elderly Chileans in the context of the COVID-19 pandemic in five regions of Chile. Int J Environ Res Public Health. (2023) 20(4):3001. 10.3390/ijerph20043001 36833696 PMC9967189

[78] Mariño R Giacaman R. Factors related to unmet oral health needs in older adults living in Chile. Arch Gerontol Geriatr. (2014) 58:454–9. 10.1016/j.archger.2014.01.003 24556393

[79] Campain A Mariño R Wright FAC Harrison D Bailey D Morgan M. The impact of changing dental needs on cost savings from fluoridation. Aust Dent J. (2010) 55:37–44. 10.1111/j.1834-7819.2010.01173.x 20415910

[80] Biblioteca del Congreso Nacional de Chile. Ley Chile—Decreto 20. Aprueba Reglamento De Establecimientos de Larga Estadía Para Personas Mayores (ELEAM). Ministerio de Salud, Subsecretaría de Salud Pública (September 30, 2022). Santiago: Biblioteca del Congreso Nacional (2022). Available online at: https://www.bcn.cl/leychile/navegar?idNorma=1182129 (Accessed March 3, 2026).

[81] Latapiat A Cornejo M Pizarro A Urzúa M. Tesis: existencia de protocolos de higiene bucal en los establecimientos de larga estadía para adultos mayores (ELEAM) y caracterización de sus directores técnicos. Universidad de Chile, Facultad de Odontología, Instituto de Investigación en Ciencias Odontológicas, Área de Salud Pública (2016). Available online at: http://repositorio.uchile.cl/bitstream/handle/2250/142506/Existencia-de-protocolos-de-higiene-bucal-en-los-establecimientos-de-larga-estad%C3%ADa.pdf?sequence=1&isAllowed=y (Accessed May 08, 2026).

[82] Corbett FC Valenzuela DS. Conocimiento y Percepción de Estudiantes Sobre Fluoruro Diamino de Plata Para Caries en Personas Mayores. Talca: Universidad de Talca (2021). Available online at: https://repositorio.utalca.cl/repositorio/server/api/core/bitstreams/f62fab77-1806-43eb-9f70-94052329f050/content (Accessed May 08, 2026).

[83] Montaña R Krashna Merino Pérez C Leiva Lagos M Fuentes Barría H. Eficacia del fluoruro de diamino de plata en el tratamiento cariostático de la primera infancia: una revisión sistemática. Universidad Andrés Bello, Facultad de Odontología (2022). Available online at: https://repositorio.unab.cl/server/api/core/bitstreams/334932d4-6748-46c0-9107-f31be148d554/content

[84] Celis A Conway DI Macpherson LMD Celis-Dooner J McMahon AD. Outcome of a national education program on supervised daily toothbrushing and biannual fluoride varnish application on dental caries in Chilean preschool children: an ecological cohort study. Caries Res. (2025) 60(2):152–62. 10.1159/000546679 40472824 PMC12227189

[85] Bridge G Martel AS Lomazzi M. Silver diamine fluoride: transforming community dental caries program. Int Dent J. (2021) 71(6):458–61. 10.1016/j.identj.2020.12.017 33653594 PMC9275183

[86] Raskin SE Tranby EP Ludwig S Okunev I Frantsve-Hawley J Boynes S. Survival of silver diamine fluoride among patients treated in community dental clinics: a naturalistic study. BMC Oral Health. (2021) 21:35. 10.1186/s12903-020-01379-x 33472613 PMC7816144

[87] da Rocha DL da Costa DB da Silva HV Silva AC de Ulhôa Santos P Rosa AC. Efficacy of silver diamine fluoride versus fluoride varnish in caries prevention and treatment in deciduous teeth. Dent Med Res. (2024) 12(2):52–7. 10.4103/dmr.dmr_16_24

[88] Gao SS Amarquaye G Arrow P Bansal K Bedi R Campus G Global oral health policies and guidelines: using silver diamine fluoride for caries control. Front Oral Health. (2021) 2:685557. 10.3389/froh.2021.685557 35048029 PMC8757897

[89] World Health Organization. Global Strategy and Action Plan on Oral Health 2023–2030. Geneva: World Health Organization (2024). Available online at: https://www.who.int/publications/m/item/who-discussion-paper-draft-global-strategy-on-oral-health (Accessed May 08, 2026).

[90] Riley JC Lennon MA Ellwood RP. The effect of water fluoridation and social inequalities on dental caries in 5-year-old children. Int J Epidemiol. (1999) 28:300–5. 10.1093/ije/28.2.300 10342695

[91] United Nations, Sustainable Development Group. Leave No one Behind (2021a). Available online at: https://unsdg.un.org/2030-agenda/universal-values/leave-no-one-behind (Accessed March 3, 2026).

[92] United Nations. The Sustainable Development Goals Report 2021. New York: United Nations (2021b). Available online at: https://unstats.un.org/sdgs/report/2021/ (Accessed March 3, 2026).

